# LncRNA NEAT1 knockdown attenuates autophagy to elevate 5‐FU sensitivity in colorectal cancer via targeting miR‐34a

**DOI:** 10.1002/cam4.2746

**Published:** 2019-12-05

**Authors:** Fen Liu, Fei‐Yan Ai, De‐Cai Zhang, Li Tian, Zhen‐Yun Yang, Shao‐Jun Liu

**Affiliations:** ^1^ Department of Gastroenterology The Third Xiangya Hospital of Central South University Changsha P.R. China; ^2^ Hunan Key Laboratory of Nonresolving Inflammation and Cancer Changsha P.R. China

**Keywords:** autophagy, colorectal carcinoma, HMGB1, LncRNA NEAT1, miR‐34a

## Abstract

**Backgrounds:**

Colorectal carcinoma (CRC) is a common malignant tumor. Increasing evidences indicated that CRC showed a resistance to 5‐fluorouracil (5‐FU) and further resulted in a poor prognosis. In this study, we aim to investigate the effect of long noncoding RNA nuclear paraspeckle assembly transcript 1 (LncRNA NEAT1) on cell viability, sensitivity to 5‐FU, and autophagy of CRC cell lines.

**Methods:**

MTT (3‐(4,5‐dimethyl‐2‐thiazolyl)‐2,5‐diphenyl‐2‐Htetrazolium bromide) was used to detect cell viability, immunofluorescent staining was used to detect autophagy puncta, and luciferase reporter system was used to determine binding ability between miR‐34a and NEAT1 or putative targets. Additionally, indicated mRNAs and protein expressions were determined by qRT‐PCR or western blotting, respectively.

**Results:**

We found that NEAT1 expression was increased in CRC tissues and cells, which showed a negative correlation with miR‐34a expression. In addition, NEAT1 knockdown noticeably inhibited the proliferation of CRC cells and enhanced 5‐FU sensitivity. It revealed that NEAT1 knockdown suppressed the LC3 puncta and the expressions of Beclin‐1, ULK1, and ratio of LC3II/I. Overexpression of miR‐34a showed similar trends with NEAT1 knockdown. miR‐34a was validated to target the putative binding sites in 3′‐UTR of HMGB1, ATG9A, and ATG4B, which are involved in the activation of autophagy. Inhibition of miR‐34a or overexpression of HMGB1 could effectively reverse elevated 5‐FU sensitivity upon NEAT1 knockdown. In addition, 3‐MA reversed NEAT1 overexpression‐induced resistance in HT29 cells.

**Conclusion:**

These findings indicate that LncRNA NEAT1 could target miR‐34a and promote autophagy to facilitate 5‐FU chemoresistance in CRC.

## Graphical abstract

NEAT1 knockdown attenuates autophagy to elevate 5‐FU sensitivity in CRC cell lines. NEAT1 is an endogenous compete RNA of miR‐34a in CRC.

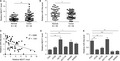

## 1 INTRODUCTION

Colorectal cancer (CRC), one of the leading cause of cancer deaths around the world, is mainly from genetic and epigenetic changes in colon epithelial cells.^1^ 5‐fluorouracil (5‐FU), a common pyrimidine analogue for CRC, could block thymidylate synthetase and further inhibit the synthesis of DNA and RNA.^2^ However, clinical treatment with 5‐FU for CRC is restricted due to its chemoresistance and results in poor prognosis.^3^ Therefore, understanding the mechanism of the resistance to 5‐FU is crucial to the chemotherapy of CRC.

Autophagy is a broad phenomenon that regulates the lysosomal degradation of proteins, ribosomes, and organelles, which is temporarily triggered by cellular stress, such as starvation, hypoxia, and metabolic disorder.^4^ Indeed, autophagy is exploited in tumor microenvironment, which helps these cancer cells adapt diverse therapies, like chemotherapy, radiation therapy, and antiestrogen hormonal therapy.^5^, ^6^ Especially, when autophagy is suppressed, the efficacy of the above therapies could be augmented.^7^

MicroRNAs (miRNAs), a group of highly conserved and small noncoding regulatory RNA molecules, perform modulatory function in many biological processes.^8^ The dysregulation of miRNA would greatly result in the development of cancer.^9^ Increasing evidences indicated that miRNAs are involved in chemoresistance of diverse cancers. In bladder cancer chemoresistance, miR‐193a‐3p targeted the expression of SRSF2, PLAU, and HIC2 and further affected their modulatory activities.^10^ Overexpression of miR‐21 aggravated the progression of colon adenocarcinomas.^11^ MiR‐34a‐5p targeted the Delta‐like ligand 1 (DLL1) gene and facilitated the multi‐chemoresistance of osteosarcoma.^12^ MiR‐1 negatively regulated the expression of stromal cell‐derived factor 1 in cancer‐associated fibroblasts and increased the sensitivity to cisplatin.^13^ MiR‐484 promoted the chemoresistance of breast cancer to gemcitabine via regulating cytidine deaminase.^14^

As a suppressor gene of tumor, miR‐34a inhibits the survival, proliferation, and invasion of cancer cells, which plays a crucial role in regulating cancer development.^15^ High mobility group box 1 (HMGB1), a conserved DNA‐binding protein that regulates the transcription of genes, is highly related to growth, invasion, and metastasis of cancer cells.^16^ HMGB1 could activate autophagy to protect cancer cell from the chemotherapy‐induced apoptosis in leukemia.^17^ miR‐34a was reported to target HMGB1 and inhibited the autophagy in acute myeloid leukemia cells.^18^ However, the mechanism of miR‐34a/HMGB1 axis in colon cancer remains unknown and needs to be further explored.

Long noncoding RNA nuclear paraspeckle assembly transcript 1 (LncRNA NEAT1), a nuclear‐restricted long noncoding RNA, modulates gene expression in nucleus and consequently regulates pathophysiological processes.^19^, ^20^ LncRNA NEAT1 promoted the proliferation and progression of human breast cancer in hypoxia.^21^ Moreover, lncRNA NEAT1 effectively inhibited the degradation of PINK1 protein and strongly suppressed MPTP‐induced autophagy in Parkinson's disease.^22^ However, whether NEAT1 could regulate the autophagy in CRC needs to be highly developed.

In present study, the expression levels of NEAT1 and miR‐34a were evaluated in CRC cell lines. Furthermore, the effects of NEAT1 and miR‐34a on cell sensitivity to 5‐FU and autophagy and its potential mechanism were determined in CRC cell lines.

## 2 MATERIALS AND METHODS

### 2.1 Cell culture

Human normal colonic epithelial cell line, FHC, and colorectal carcinoma cell lines, HT29, HCT8, HCT116, SW480, SW620, were purchased from ATCC and cultured in DMEM medium which contained 10% fetal bovine serum and 1% penicillin‐streptomycin with 5% CO_2_ at 37°C.

### 2.2 Patient and tissue samples

Colorectal tissue and respective adjacent nontumor tissues were obtained from 55 patients who received resection of primary CRC between 2015 and 2017 at the Third Xiangya Hospital, Central South University (Changsha, China). Histopathological examination was applied to determine samples. Based on the exclusion criteria, patients who received no preoperative treatment, including radiotherapy or chemotherapy, were enrolled. All patients were informed and understood the procedure and provided written informed consent before research. The present study was approved by the Ethics Committee of Central South University.

### 2.3 Transfections

HCT8 and SW480 cells (1 × 10^5^ cells) were seeded in 6‐well plates in DMEM medium for 12 hours. Then, cells were transfected with 10 nmol/L mimic nontargeting control and the relevant miRNA mimic, and 10 nmol/L inhibitor nontargeting control and the relevant miRNA inhibitor through Oligofectamine transfection reagent. Next, HCT8 and SW480 cells (1 × 10^5^ cells) were plated in 6‐well plates and transfected with 5 nmol/L NEAT1 shRNA or shRNA negative control via 0.2% Lipofectamine 3000 in DMEM medium. The synthetic miR‐34a mimic, miR‐34a inhibitor and their negative control (miR‐control), specific shRNA targeting NEAT1 or scramble shRNA, and human HMGB1 overexpressing plasmid were obtained from GenePharma. After 5 hours, the transfection reagent was replaced with DMEM medium. Forty‐eight hours later, cells were collected for further experiments.

### 2.4 Cell viability

Cell viability was determined by MTT (3‐(4,5‐dimethyl‐2‐thiazolyl)‐2,5‐diphenyl‐2‐H‐tetrazolium bromide) assay. HCT8 and SW480 cells (1 × 10^5^ cells/well) were seeded in 96‐well plates. After 12 hours, different concentrations of 5‐FU were added and incubated for further 24 hours. Solvent was added as negative control group. Then, each well was incubated with MTT (0.2 mg/mL) at 37°C for 4 hours. Supernatants were discarded and 100 μL DMSO was added to dissolve the formazan crystals. The absorbance at 490 nm was measured with a microplate reader. The relative viability of treated cells was expressed as percentage of control cells.

### 2.5 Colony formation test

HCT8 and SW480 cells (0.5 × 10^3^ cells/mL) were seeded in 6‐well plates in DMEM medium at 37°C. Half of medium was replenished on day 5 and the medium was discarded on day 14. Cells were washed with phosphate‐buffered saline, fixed with methanol for 10 min, and stained with crystal violet for 5 minutes. Thereafter, cells were observed under a microscope and five fields were selected for colony counting.

### 2.6 Western blotting

Cells were collected and lysed with RIPA lysis buffer (Zhong‐Shan Jin Qiao) for 30 minutes. Protein concentrations were determined by BCA kits. Equal amounts of total proteins (25 μg) were determined by electrophoresis with 10% SDS‐PAGE. Subsequently, proteins were transferred onto polyvinylidene difluoride membranes (PVDF, MA, USA). The PVDF membranes were blocked with 5% milk for 1 hour and then incubated with primary antibodies (LC3B antibody, Beclin‐1 antibody, ULK1 antibody, and ATG5 antibody) (1:1000; Cell Signaling Technology) and β‐actin (1:3000; Cell Signaling Technology) at 4°C for 16 hours. Thereafter, the membranes were incubated with the appropriate horseradish peroxidase‐conjugated secondary antibody. The protein bands were observed by chemiluminescence via the ECL reagents. Quantification of protein bands was conducted by ImageJ Software. Statistical analysis was done based on at least three bands from independent experiments. Relative protein level was determined as the ratio of individual protein gray level and their β‐actin gray level.

### 2.7 Quantitative RT‐PCR

Total RNA was extracted using Trizol reagents and the quality of RNA was determined with an Agilent 2100 Bioanalyzer (Agilent Technologies). Whole RNA (500 ng) was reversely transcribed into cDNA using miRNA‐specific TaqMan miRNA assay kit (Applied Biosystems). The expression levels of lncRNA NEAT and miR‐34a were quantified using SYBR^®^ Premix Ex TaqTMII (Perfect Real Time) on a CFX 96 real‐time PCR thermocycler. GAPDH and U6 were used as the internal control. Relative amounts were expressed as the 2^−ΔΔCt^. The reaction conditions of qRT‐PCR were as follows: 92°C for 10 minutes, then 40 cycles at 92°C for 10 seconds and 60°C for 1 minute. The primer sets used in the present study were shown as follows: NEAT1 forward 5′‐GTGGCTGTTGGAGTCGGTAT‐3′ and reverse 5′‐TAACAAACCACGGTCCATGA‐3′. GAPDH forward 5′‐CCAGGTGGTCTCCTCTGA‐3′ and reverse 5′‐GCTGTAGCCAAATCGTTGT‐3′. miR‐34a forward 5′‐CCGTGGCAGTGTCTTAGCT‐3′ and reverse 5′‐CGGCCCAGTGTTCAGACTAC‐3′. U6 forward 5′‐CTCGCTTCGGCAGCACA‐3′ and reverse 5′‐AACGCTTCACGAATTTGCGT‐3′.

### 2.8 Immunofluorescence staining

Initially, cells were fixed with 4% paraformaldehyde for 10 minutes, followed by permeabilization with 0.02% Triton X‐100. Cells were blocked with 5% bovine serum albumin in PBS. Then, 50 μg/mL LC3 primary antibody was incubated with the cells at 4°C. After 12 hours, cells were washed with PBS for three times, and TRITC‐conjugated secondary antibody was incubated with the cells at room temperature for 2 hours. Negative control was in the absence of primary antibody. The images were observed using a charge‐coupled device‐equipped photomicroscope (Olympus IX71). The number of LC3 puncta in the cells was recorded and quantitated by ImageJ software. A minimum of 50 cells were counted for each group.

### 2.9 Dual Luciferase assay

The fragments, 3′‐UTRs of HMGB1, ATG9A, and ATG4B contained the wild‐type (wt) and the mutant (mut) binding sites of miR‐34a, were cloned into the downstream of firefly luciferase coding gene in the pMIR‐Report vector (Ambion Inc). The constructed luciferase reporters were called as pMIR‐LUC‐3′‐UTR‐WT HMGB1 and pMIR‐LUC‐3′‐UTR‐HMGB1‐MUT, pMIR‐LUC‐3′‐UTR‐WT ATG9A and pMIR‐LUC‐3′‐UTR‐ATG9A‐MUT, and pMIR‐LUC‐3′‐UTR‐WT ATG4B and pMIR‐LUC‐3′‐UTR‐ATG4B‐MUT. For luciferase assay, HCT8 cells (1 × 10^5^ cells/well) were seeded in 24‐well plates, miR‐34a mimic or mimic NC (100 nmol/L) was co‐transfected with reporter plasmids (100 ng) using Lipofectamine 3000. After 48 hours, the luciferase activities were measured using the Dual Luciferase kit (Promega). Cells transfected with 100 ng pMIR‐control vector were used as blank and Renilla luciferase was used as control.

### 2.10 Statistical analysis

Each experiment was repeated at three independent times. Data were presented as the mean ± SD. Unpaired two‐tailed Student's *t* test between two groups and one‐way ANOVA followed by Tukey's post hoc test between multiple groups were applied. Prism software version 6 (GraphPad software) was used to plot. Differences were considered as significant where *P* < .05 represented as * and *P* < .01 represented as **.

## 3 RESULTS

### 3.1 Differential expression of NEAT1 and miR‐34a in CRC cell lines and tissue

**Figure 1:**
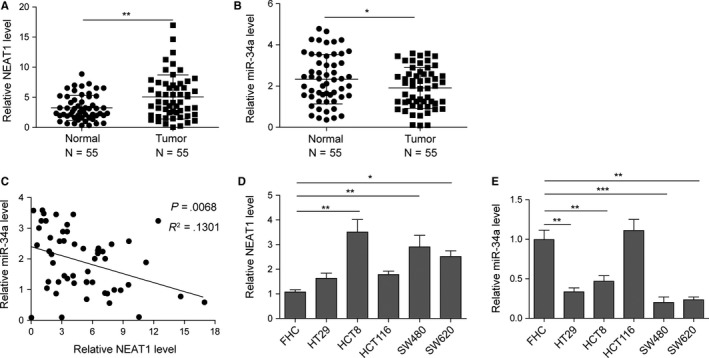
Expression of NEAT1 and miR‐34a in colorectal carcinoma (CRC) cell lines and tissue. A and B, Relative NEAT1 and miR‐34a levels in CRC tissue and adjacent tissue were determined by qRT‐PCR (n = 55). C, The relationship between NEAT1 and miR‐34a in CRC tissue analyzed by Pearson's correlation analysis. D and E, Relative NEAT1 and miR‐34a levels in CRC cell lines and normal cell lines were determined by qRT‐PCR. Data were pooled from at least three independent experiments; *P < .05, **P < .01 and ***P < .001

The differential expressions of NEAT1 and miR‐34a in CRC tissue and adjacent nontumor tissue were investigated using qRT‐PCR method. The results revealed that NEAT1 expression in CRC tissue was strongly higher than that in adjacent tissue (Figure 1A, *P* < .01), while miR‐34a expression showed opposite trends (Figure 1B), indicating that NEAT1 might participate in CRC pathogenesis. In addition, Pearson's correlation analyzes showed a significant negative correlation between NEAT1 expression and miR‐34a expression (Figure 1C). Next, we determined the expression of NEAT1 in FHC, HT29, HCT8, HCT116, SW480, and SW620 cell lines. The results revealed that NEAT1 mRNA expression in CRC cell lines was higher than that in normal cell line (FHC), while the miR‐34a expression in CRC cell lines was greatly lower than that in FHC (Figure 1E). To investigate the mechanism of NEAT1 in CRC, HCT8 and SW480 cell lines were selected for the following experiments.

### 3.2 NEAT1 knockdown downregulated the proliferation and elevated sensitivity to 5‐FU of HCT8 and SW480

**Figure 2:**
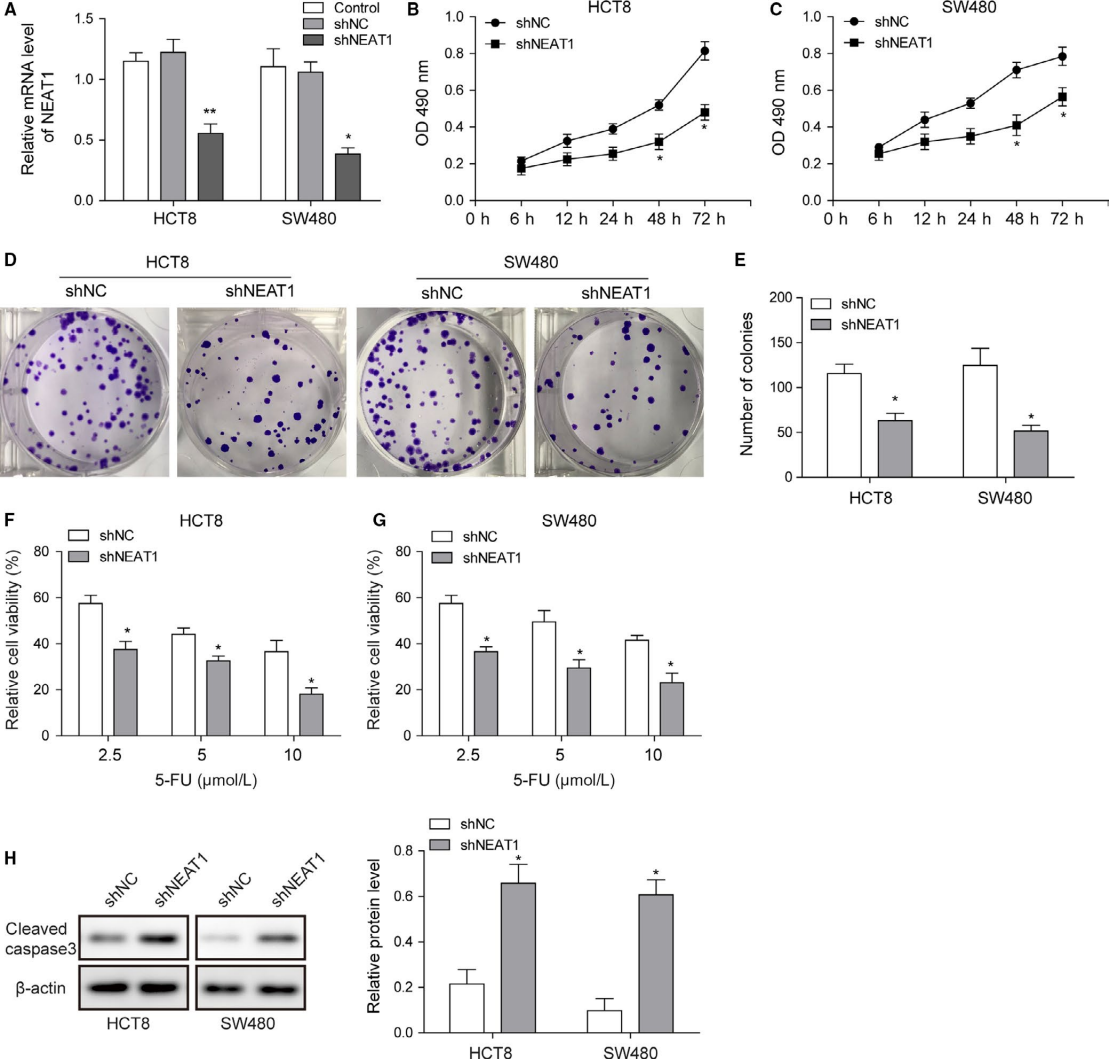
NEAT1 knockdown downregulated the proliferation and elevated sensitivity to 5‐fluorouracil (5‐FU) of HCT8 and SW480. A, Relative NEAT1 levels in colorectal carcinoma (CRC) cell lines determined by qRT‐PCR. B and C, MTT results of HCT8 and SW480 cells when treated with shRNA NEAT1. D and E, Colony formation of HCT8 and SW480 cells when treated with shRNA NEAT1. F and G, The sensitivity to 5‐FU of HCT8 and SW480 cells when treated with shRNA NEAT1. H, Representative image of cleaved caspase‐3 in HCT8 and SW480 cells when treated with shRNA NEAT1 was determined by western blotting and quantitative analysis of relative protein level. Data were pooled from at least three independent experiments; *P < .05 and **P < .01

Expression of NEAT1 in HCT8 and SW480 cell lines transfected with shRNA NEAT1 was confirmed using qRT‐PCR. Compared with shRNA negative control group, shRNA NEAT1 group significantly decreased the NEAT1 expression in HCT8 and SW480 cell lines (Figure 2A), whereas shRNA negative control group showed no significant difference in NEAT1 expression with control group (Figure 2A). MTT assay showed that at 48 and 72 hours, shRNA NEAT1 remarkably reduced cell viability compared with shRNA negative control group in HCT8 and SW480 cell lines (Figure 2B,C). Colony formation results showed that shRNA NEAT1 inhibited the proliferation of HCT8 and SW480 cells (Figure 2D,E). When treated with different dosage of 5‐FU in HCT8 and SW480 cell lines, shRNA NEAT1 group increased sensitivity to 5‐FU than shRNA negative control group (Figure 2F,G). Western blot results indicated that shRNA NEAT1 also increased the expression of cleaved caspase‐3, which is an apoptotic marker, in HCT8 and SW480 cells (Figure 2H).

### 3.3 NEAT1 knockdown suppressed autophagy in HCT8 and SW480 via miR‐34a

**Figure 3:**
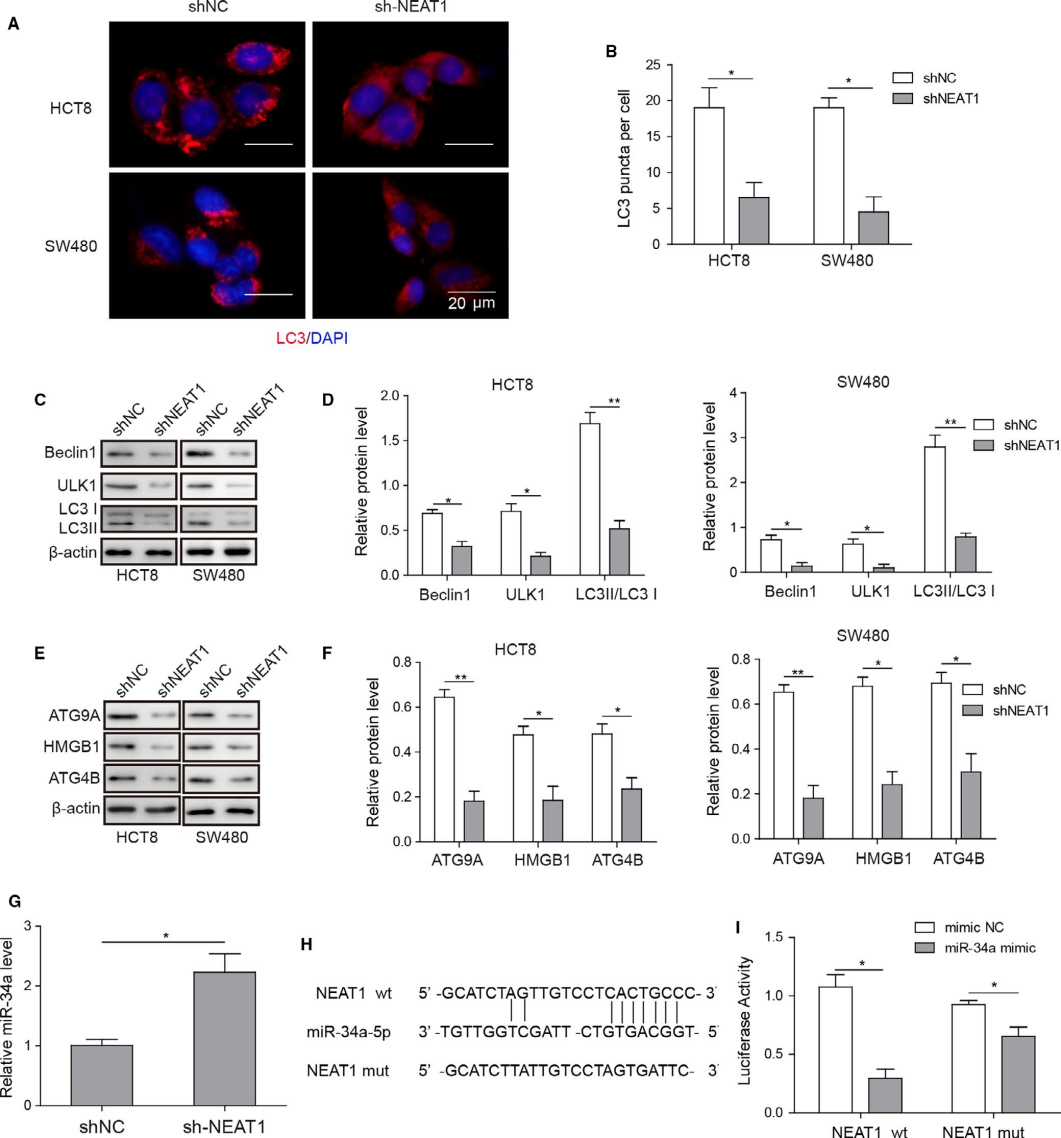
NEAT1 knockdown attenuated autophagy via miR‐34a in HCT8 and SW480. A and B, Fluorescent images of LC3 puncta in colorectal carcinoma (CRC) cell lines and quantitative analysis of LC3 puncta per cell. C and D, Representative image of LC3 II/I, Beclin‐1, and ULK1 in HCT8 and SW480 cells transfected with shRNA NEAT1 was determined by western blotting and quantitative analysis of relative protein level. E and F, Representative image of ATG9A, ATG4B, and HMGB1 in HCT8 and SW480 cells transfected with shRNA NEAT1 was determined by western blotting and quantitative analysis of relative protein level. G, Relative miR‐34a levels in CRC cell lines determined by qRT‐PCR. H, Illustration of prediction of miR‐34a binding sites on target gene NEAT1. I, Dual luciferase assays were carried out after HCT8 were co‐transfected pMIR‐LUC‐3′‐UTR‐NEAT1‐wt or pMIR‐LUC‐3′‐UTR‐NEAT1‐mut and miR‐34a mimic or NC for 48 h. Data were pooled from at least three independent experiments; *P < .05 and **P < .01

Next, we determined the effect of shRNA NEAT1 on the formation of autophagy puncta using immunofluorescent staining. The results revealed that the number of LC3 puncta in shRNA NEAT1 group was evidently lower than that in shRNA negative control group (Figure 3A,B). Autophagy‐related proteins were determined by western blotting. The results showed that NEAT1 knockdown inhibited the protein expression of Beclin‐1 and ULK1 and lowered the ratio of LC3II/I in HCT8 and SW480 cell lines (Figure 3C,D). Moreover, we determined the expression of HMGB1 and autophagy‐related proteins ATG9A and ATG4B. The results demonstrated that shRNA NEAT1 group reduced protein expression of ATG9A, ATG4B, and HMGB1 in HCT8 and SW480 cell lines (Figure 3E,F). We also explored the effect of NEAT1 knockdown on the expression of miR‐34a in HCT8 and SW480 cell lines. shRNA NEAT1 group greatly increased the expression of miR‐34a (Figure 3G). The binding between NEAT1 and miR‐34a was determined by luciferase assay. In NEAT1 WT group, compared with mimic NC, miR‐34a mimic obviously reduced luciferase activity. However, in NEAT1 mutant group, there was no difference in luciferase activity between mimic NC and miR‐34a mimic group (Figure 3H,I).

### 3.4 Overexpression of miR‐34a downregulated the proliferation and elevated sensitivity to 5‐FU of HCT8 and SW480

**Figure 4:**
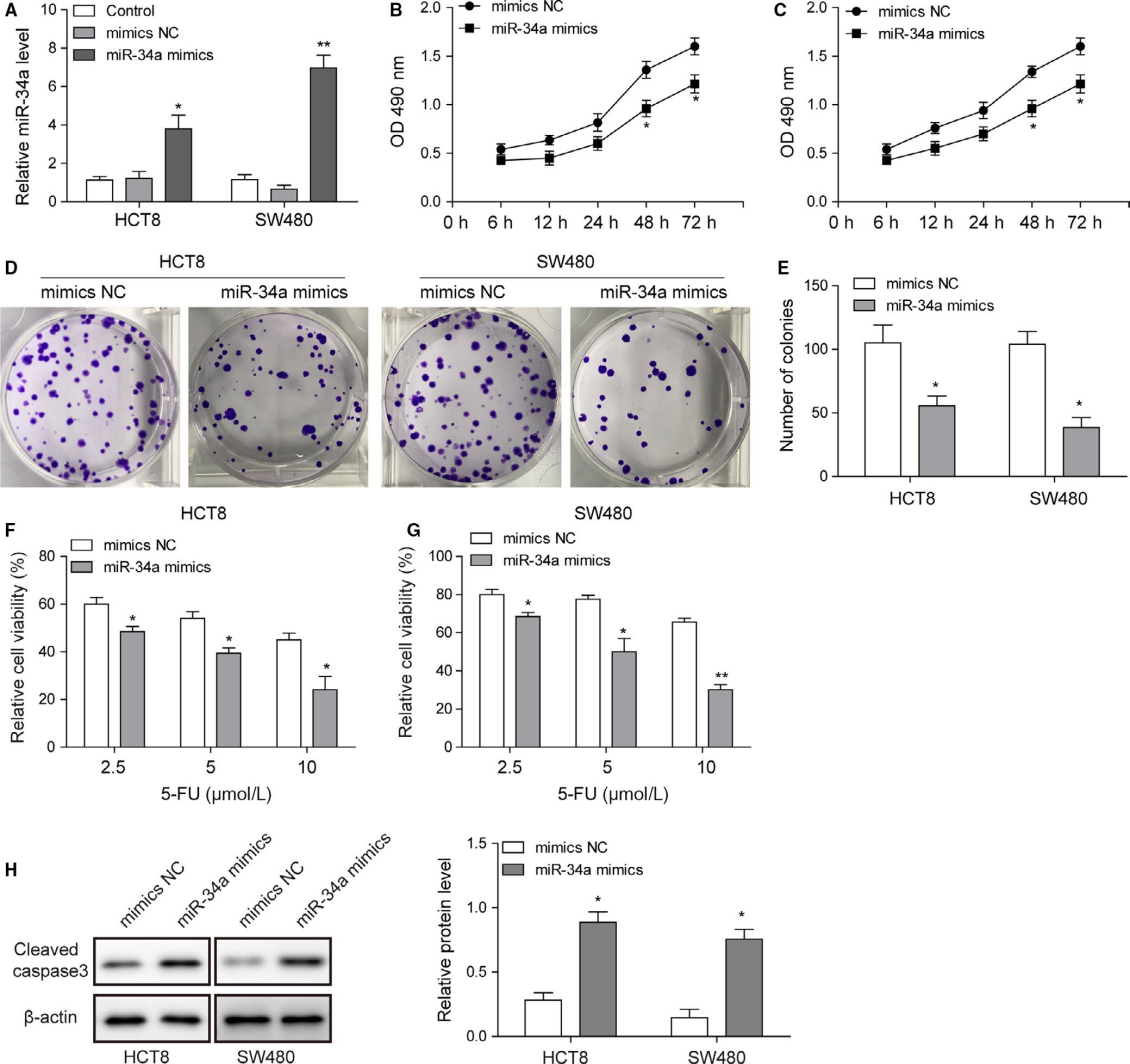
Overexpression of miR‐34a downregulated the proliferation and elevated sensitivity to 5‐fluorouracil (5‐FU) of HCT8 and SW480. A, qRT‐PCR was used to evaluate the miR‐34a expression in HCT8 and SW480 cells transfected with miR‐34a mimics and mimic NC. B and C, MTT results of HCT8 and SW480 cells transfected with miR‐34a mimic or mimic NC. D and E, Colony formation of HCT8 and SW480 cells transfected with miR‐34a mimic or mimic NC. F and G, The sensitivity to 5‐FU of HCT8 and SW480 cells transfected treated with miR‐34a mimic or mimic NC. H, Representative image of cleaved caspase‐3 in HCT8 and SW480 cells transfected with miR‐34a mimic or mimic NC was determined by western blotting and quantitative analysis of relative protein level. Data were pooled from at least three independent experiments; *P < .05 and **P < .01

Overexpression of miR‐34a in HCT8 and SW480 cell lines was validated by qRT‐PCR method. The expression level of miR‐34a in miR‐34a mimic was obviously higher than that in mimic negative control (Figure 4A). Moreover, cell viability of miR‐34a mimic group was lower than that of mimic negative control group at 48 and 72 hours (Figure 4B,C). Colony formation results showed that miR‐34a mimic inhibited the proliferation of HCT8 and SW480 cells (Figure 4D,E). Additionally, compared with mimic negative control, miR‐34a mimic elevated the sensitivity to 5‐FU of HCT8 and SW480 cell lines (Figure 4F,G). Western blots results indicated that miR‐34a mimic highly increased the expression of cleaved caspase‐3 in HCT8 and SW480 cells (Figure 4H).

### 3.5 Overexpression of miR‐34a inhibited autophagy and its related proteins in HCT8 and SW480

**Figure 5:**
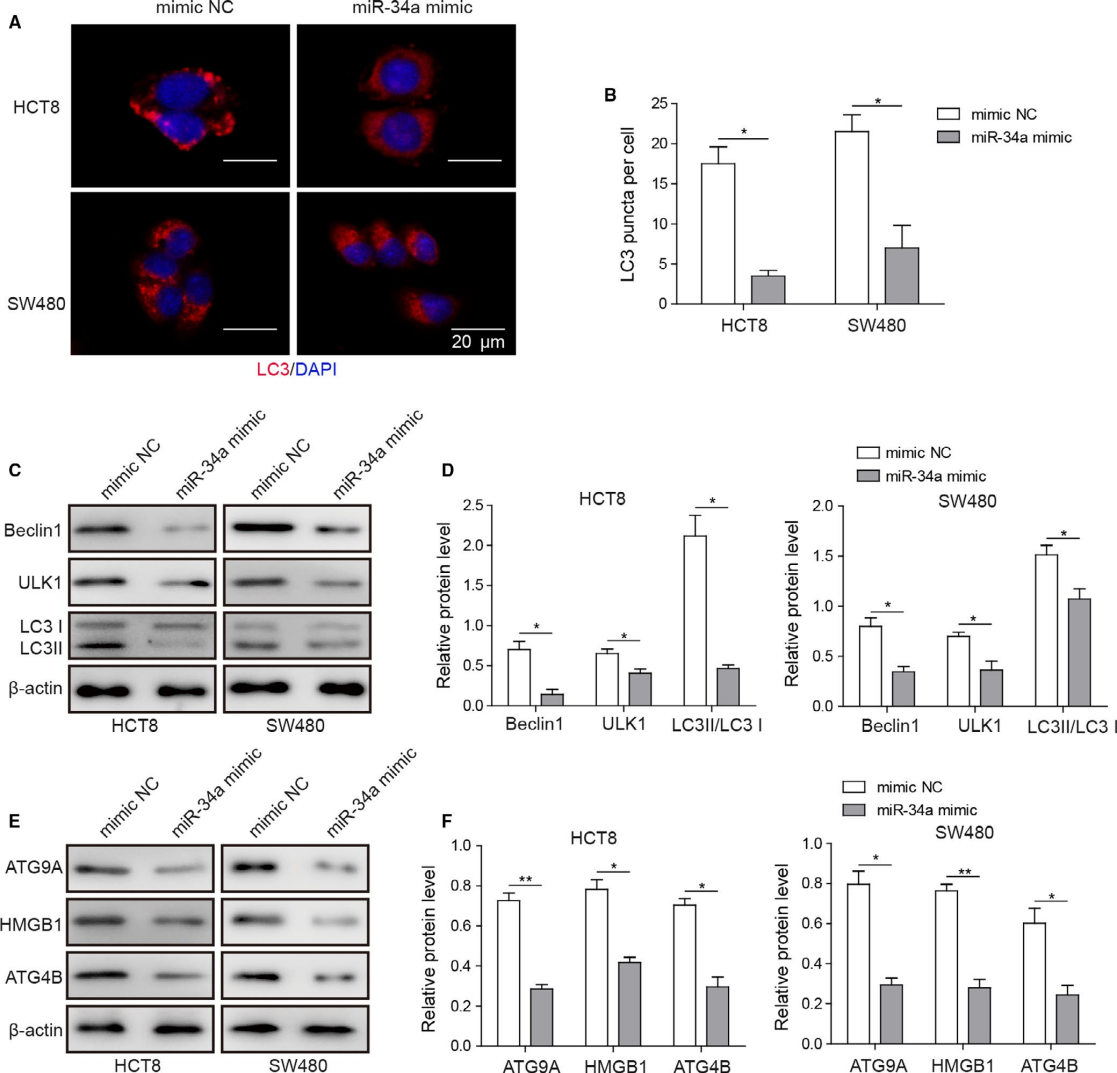
Overexpression of miR‐34a inhibited autophagy and its related proteins in HCT8 and SW480. A and B, Fluorescent images of LC3 puncta in colorectal carcinoma cell lines and quantitative analysis of LC3 puncta per cell. C and D, Representative image of LC3II/I, Beclin‐1, and ULK1 in HCT8 and SW480 cells transfected with miR‐34a mimic was determined by western blotting and quantitative analysis of relative protein level. E and F, Representative image of ATG9A, ATG4B, and HMGB1 in HCT8 and SW480 cells transfected with miR‐34a mimic were determined by western blotting and quantitative analysis of relative protein level. Data were pooled from at least three independent experiments; *P < .05 and **P < .01

The effect of miR‐34a mimic on the formation of autophagy puncta was determined by immunofluorescent staining. Overexpression of miR‐34a decreased the number of LC3 puncta when compared with mimic negative control (Figure 5A,B). The autophagy‐related proteins were determined by western blotting. miR‐34a mimic reduced the protein expression of Beclin‐1 and ULK1 and the ratio of LC3II/I in HCT8 and SW480 cell lines (Figure 5C,D). In addition, expression of miR‐34a putative targets, which were involved in autophagy, was evaluated using western blotting. Results showed that miR‐34a mimic suppressed the protein expression of ATG9A, ATG4B, and HMGB1 in HCT8 and SW480 cell lines (Figure 5E,F).

### 3.6 miR‐34a targeted HMGB1, ATG9A, and ATG4B

**Figure 6:**
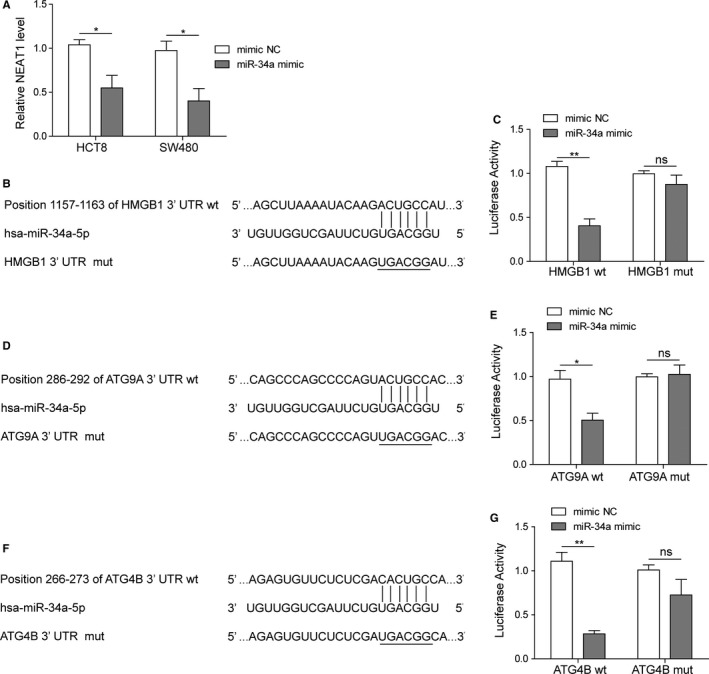
miR‐34a targeted HMGB1, ATG9A, and ATG4B. A, Relative NEAT1 levels in colorectal carcinoma (CRC) cell lines determined by qRT‐PCR. B, Illustration of prediction of miR‐34a binding sites on target gene HMGB1 by Targetscan. C, Dual luciferase assays were carried out after CRC cell lines were co‐transfected pMIR‐LUC‐3′‐UTR‐HMGB1‐wt or pMIR‐LUC‐3′‐UTR‐mut and miR‐34a mimic or NC HMGB1‐mut for 48 h. D, Illustration of prediction of miR‐34a binding sites on target gene ATG9A by Targetscan. E, Dual luciferase assays were carried out after CRC cell lines were co‐transfected pMIR‐LUC‐3′‐UTR‐ATG9A‐wt or pMIR‐LUC‐3′‐UTR‐ATG9A‐mut and miR‐34a mimic or NC for 48 h and miR‐34a mimic or NC. F, Illustration of prediction of miR‐34a binding sites on target gene ATG4B by Targetscan. G, Dual luciferase assays were carried out after CRC cell lines were co‐transfected pMIR‐LUC‐3′‐UTR‐ATG4B‐wt or pMIR‐LUC‐3′‐UTR‐ATG4B‐mut and miR‐34a mimic or NC for 48 h. Data were pooled from at least three independent experiments; *P < .05 and **P < .01

Moreover, qRT‐PCR results showed that overexpression of miR‐34a could reciprocally inhibit the expression of NEAT1 in HCT8 and SW480 cell lines (Figure 6A). To validate the interaction between miR‐34a and three putative targets, the miR‐34a‐binding region was mutated in 3′‐UTR of HMGB1, ATG9A, and ATG4B (Figure 6B,D,F). Subsequently, modified luciferase report vectors were co‐transfected with miR‐34a mimic or mimic NC. Results showed that miR‐34a mimic has no effect on luciferase activity of mutated groups while significantly decreased the luciferase activity among wild‐type groups when compared to mimic NC group (Figure 6C,E,G), indicating that the potential binding abilities between miR‐34a and putative site of HMGB1, ATG9A, and ATG4B were existed.

### 3.7 NEAT1 affected proliferation and autophagy in HCT8 and SW480 dependent on miR‐34a/HMGB1 axis

**Figure 7:**
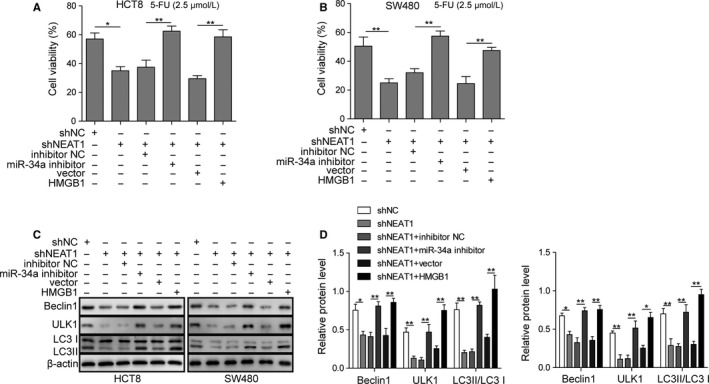
NEAT1 affected proliferation and autophagy in HCT8 and SW480 dependent on miR‐34a/HMGB1 axis. A and B, The 5‐fluorouracil (5‐FU) sensitivity of HCT8 and SW480 cells co‐transfected with indicated plasmids or nucleic acids. C and D, Representative image of LC3II/I, Beclin‐1, and ULK1 in HCT8 and SW480 cells co‐transfected with indicated plasmids or nucleic acids was determined by western blotting and quantitative analysis of relative protein level. Data were pooled from at least three independent experiments; *P < .05 and **P < .01

To prove that NEAT1 depends on miR‐34a/HMGB1 regulated autophagy and chemoresistance, we applied co‐transfection assay. Silence of miR‐34a or overexpression of HMGB1 in HCT8 and SW480 cell lines could effectively reverse the elevated 5‐FU (2.5 μmol/L) sensitivity induced by NEAT1 knockdown (Figure 7A,B). Moreover, silence of miR‐34a or overexpression of HMGB1 in HCT8 and SW480 cell lines also compromised the inhibition of autophagic markers induced by NEAT1 knockdown (Figure 7C,D).

### 3.8 Overexpression of NEAT1 increased resistance to 5‐fluorouracil (5‐FU) via inducing autophagy in HT29 cells

**Figure 8:**
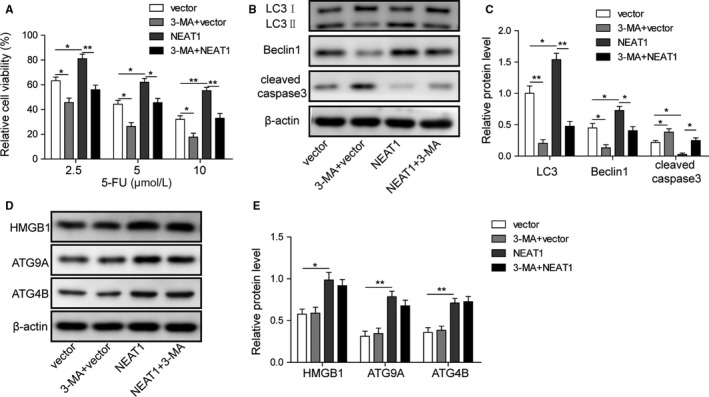
Overexpression of NEAT1 increased resistance to 5‐fluorouracil (5‐FU) was dependent on autophagy in HT29 cells. A, MTT assays were carried out when overexpression of NEAT1 and addition of 3‐MA. B and C, Representative image of LC3II/I, Beclin‐1, and cleaved caspase‐3 in HT29 cells was determined by western blotting and quantitative analysis of relative protein level. D and E, Representative image of ATG9A, ATG4B, and HMGB1 in HT29 cells was determined by western blotting and quantitative analysis of relative protein level. Data were pooled from at least three independent experiments; *P < .05 and **P < .01

Rescue experiment applied to validate NEAT1‐related 5‐FU sensitivity is dependent on regulation of autophagy. MTT assays demonstrated that overexpression of NEAT1 increased the resistance to 5‐FU in HT29 cells, in which NEAT1 has relative low expression. However, addition of 3‐MA could reverse this effect (Figure 8A). In addition, overexpression of NEAT1 activated autophagy via increasing the expression of the ratio of LC3II/I and Beclin‐1 and decreasing the expression of cleaved caspase‐3 in HT29 cells, while addition of 3‐MA comprised the change of protein profile (Figure 8B,C). Moreover, overexpression of NEAT1 upregulated the expression of HMGB1, ATG9A, and ATG4B in HT29 cells; however, supplement of 3‐MA showed no effects on them (Figure 8D,E). The results also showed that 3‐MA alone increased the sensitivity to 5‐FU in HT29 cells (Figure 8A).

## 4 DISCUSSION

In the present study, we observed high expression of NEAT1 and low expression of miR‐34a in CRC tissue. We also found that NEAT1 knockdown or miR‐34a overexpression remarkably suppressed the growth and enhanced the sensitivity to 5‐FU of CRC cell lines; further investigation disclosed that NEAT1 facilitated autophagy in CRC cell lines via downregulating miR‐34a and thus depressing miR‐34a putative targets which were involved in autophagy.

Several previous studies reported the role of NEAT1 in CRC. However, the machanism by which NEAT1 regulated the chemoresistance of CRC remained elusive.^23^ In the present study, we initially observed that NEAT1 promoted 5‐FU chemoresistance of CRC. It was reported that NEAT1 facilitated 5‐FU resistance of breast cancer via miR‐211/HMGA2 axis.^24^ In addition, NEAT1 increased 5‐FU chemoresistance of renal cell carcinoma through miR‐34a/c‐Met axis.^25^ These results indicated that NEAT1 might regulate chemoresistance of CRC via multiple ways. It was also demonstrated that autophagy modulated chemoresistance of glioblastoma through balance of mitochondrial bioenergetics,^26^ which indicated the important role of autophagy in chemoresistance; NEAT1 bound with PINK1 protein and promoted the autophagy in MPTP‐induced Parkinson's disease.^22^ Silence of NEAT1 showed a protective effect on MPTP‐induced Parkinson's mice.^27^ However, whether NEAT1 regulated autophagy in cancer was elusive. Mechanically, we firstly observed that NEAT1 regulated autophagy of CRC, which revealed a novel pathway by which NEAT1 regulated chemoresistance. Particularly, we found that NEAT1 targeted miR‐34a, depressed downstream targets of miR‐34a, and thus facilitated the protective autophagy and tumorigenesis. It was consistent with the recent reports in CRC that NEAT1 targeted miR‐34a to upregulate SIRT1/Wnt/β‐catenin axis.^28^ This suggested that lncRNA served as a ceRNA of miRNA to regulate multiple targets. In addition to chemoresistance, autophagy is also involved in many other phenotypes of cancer. In hypoxic region, basal autophagy was upregulated to facilitate survival of tumor.^29^ Meanwhile, autophagy‐regulated glioma‐initiating cells' self‐renewal and suppressed tumorigenicity depended on Notch1 degradation.^30^ Thus, our further investigation should focus on whether NEAT1 could regulate other progress in cancers via modulating autophagy.

MiR‐34a, one of the first identified tumor suppressor genes, commonly shows low expression in many tumors, such as breast cancer, lung cancer, and acute myeloid leukemia.^31^ MiR‐34a was involved in many cellular progress, like p53‐induced cell cycle arrest, apoptosis, and negatively regulated SIRT1.^32^ In the present study, NEAT1 acted as ceRNA of miR‐34a and further resulted in the elevation of downstream targets. This finding was consistent with previous report that NEAT1 was a competitive sponge for miR‐34a and prevented the inhibition of c‐Met, resulting in the increase of chemoresistance.^25^ Meanwhile, we found that miR‐34a could simultaneously target HMGB1, ATG9A, and ATG4B and finally repressed autophagy, indicating that miR‐34a manipulated single progress via targeting multiple targets. It was reported that NEAT1 was able to interact with the G9a‐DNMT1‐Snail complex and suppressed the expression of E‐cadherin in osteosarcoma.^33^ These reports indicated that NEAT1 might regulate miR‐34a expression through DNA methylation besides direct binding,^34^ which needs future investigation to prove it.

## 5 CONCLUSIONS

Our results indicated that NEAT1 was highly expressed in CRC tissue and cell lines, which was negatively with miR‐34a expression. Moreover, NEAT1 knockdown enhanced the sensitivity to 5‐FU of CRC cells. Mechanically, NEAT1 knockdown targeted miR‐34a, thus suppressed autophagy. Therefore, 5‐FU chemoresistance of CRC might be partly contributed to NEAT1 that could target miR‐34a/HMGB1/ATG9A/ATG4B axis, which could be a promising target for CRC therapy.

## CONFLICT OF INTEREST

The authors state no conflict of interest.

## Data Availability

The authors confirm that the data supporting the findings of this study are available within the article [and/or] its supplementary materials.
