# Supplementary figures and images for: LncRNA NEAT1 knockdown attenuates autophagy to elevate 5‐FU sensitivity in colorectal cancer via targeting miR‐34a
Source: Cancer Med. 2019 Dec 5;9(3):1079–91. doi: 10.1002/cam4.2746 (PMC6997058; doi:10.1002/cam4.2746)

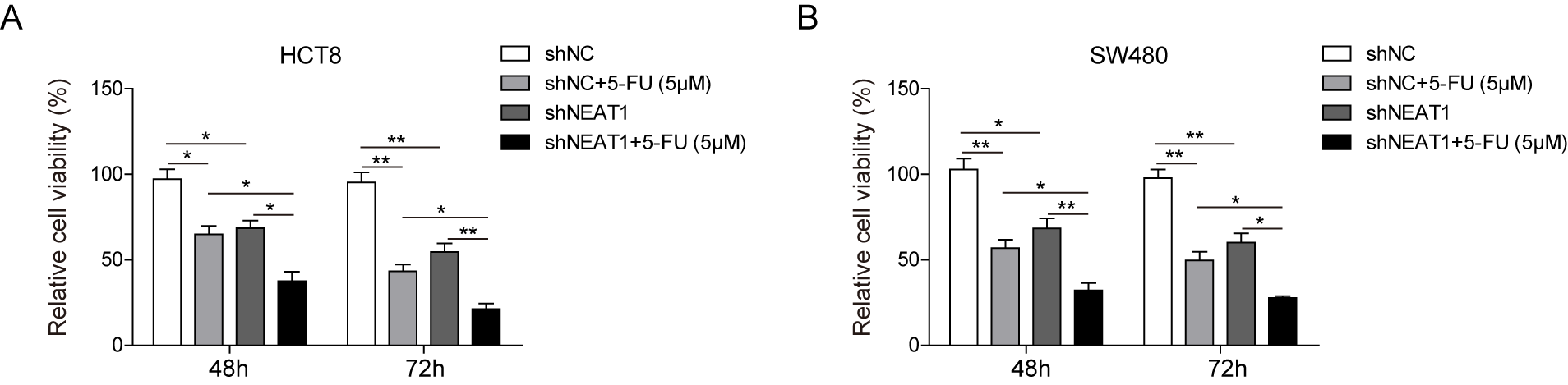

Supplement: Supplementary file 1 [file CAM4-9-1079-s001.tif]
